# Spiritual Themes and Challenges in Global Health

**DOI:** 10.1007/s10912-015-9378-9

**Published:** 2015-12-30

**Authors:** David G. Addiss

**Affiliations:** 1 Center for Compassion and Global Health, 1120 Clifton Rd, NE, Atlanta, GA 30307 USA; 2 Task Force for Global Health, 325 Swanton Way, Decatur, Georgia 30030 USA

**Keywords:** compassion, spirituality, global health, public health

## Abstract

Although the importance of spirituality is increasingly recognized in clinical medicine, spirituality is rarely mentioned in the practice, literature, or training programs of global health. To understand the role of spirituality in global health practice and identify factors that influence and limit its expression, I initiated conversations and informal interviews with more than 300 global health leaders, students, and practitioners during 2010-2014. Four spiritual themes or challenges emerged: compassion at a distance; dichotomous thinking; conspiracy of silence; and compulsion to save the world. Practitioners expressed strong interest in bringing spirituality more fully into global health discourse, which could help the field realize its potential.

## Introduction

The field of medicine is grounded in the ultimate questions of health and wholeness, of meaning and purpose in both life and death (Fricchione 2011). These matters are also central to spirituality. A 2009 National Consensus Conference, *Improving the Quality of Spiritual Care as a Dimension of Palliative Care,* defined spirituality as “that aspect of humanity that refers to the way individuals seek and express meaning and purpose and experience their connectedness to the moment, to self, to others, to nature, and to the significant or sacred” (Puchalski 2009).

The critical role of spirituality in medicine and health care has been increasingly recognized in recent years (Puchalski 2014) with palliative care (Edwards 2010; Rumbold 2003) and nursing (Lewinson 2015; Rudolfsson 2014) at the forefront. Addressing spiritual issues is associated with improved health outcomes and enhanced quality of life for patients with mental illness (Moreira-Almeida 2014), heart disease (Naghi 2010), cancer (Piderman 2015), and other diseases. Spiritual concerns are among the most pressing for patients facing catastrophic illness and end of life, as well as for their family and caregivers (Fricchione 2011).

The importance of spirituality for health care providers is also receiving greater attention. Almost sixty years ago, Abraham Heschel reminded delegates to the American Medical Association convention that, “to heal a person one must first be a person” (Sulmasy 2006). In addition to enhancing physicians’ capacity to perceive and address the spiritual needs of their patients, spirituality offers coping strategies to deal with stress and loss as well as protection against burnout (Doolittle 2013). Consequently, several major medical institutions have established centers to study and promote spirituality in health care (Puchalski and Ferrell 2010) and standards have been proposed for spirituality in medical education and clinical practice (Puchalski 2014).

In sharp contrast, spirituality is rarely mentioned in the practice, literature, or training programs of the rapidly growing field of global health. Global health emerged during the 1990s from the disciplines of tropical medicine, public health, and international health as a response to novel infectious disease threats, globalization of the economy, the environmental movement, and new public health partnerships and alliances (Brown et al. 2006). The field of global health is characterized by its worldwide geographic reach, its emphasis on multidisciplinary collaboration and multilateral coordination, and its embrace of both clinical care and prevention (Koplan 2009; Beaglehole and Bonita 2010). Rooted in an ethic of social justice, global health aims to improve health and achieve health equity for *all* people (Koplan 2009).

In its principles, if not always in practice, global health is unabashedly non-sectarian. The inscription on a granite wall in the lobby of the World Health Organization (WHO) in Geneva points to global health’s extraordinary, radical proposition: “the attainment by *all* peoples of the highest possible level of health.” This aspiration extends to those who live in countries that my government – and yours – consider as enemies. Global health is grounded in a deep sense of human interconnectedness, which transcends barriers of race, religion, economics, and nationality (Addiss, in press). The universal language that global health uses to describe itself testifies to its rootedness in the spirituality of compassion – which is also proclaimed by the great religious traditions (Armstrong 2004).

Global health often traces its origins to ‘missionary medicine’ of the 19^th^ and early 20^th^ centuries, which expressed its spiritual vision and purpose in distinctly religious, primarily evangelical Christian, terms (Brown 2014). By religion I mean the communal tradition that contains, passes along, and holds the space for a certain experience of the sacred, comprised of shared beliefs, practices, texts, and conceptual frameworks. The relationship between religious institutions and the spirituality that (at its best) religion nurtures is inherently complex. Journalist Krista Tippet, in a recent interview with Pico Iyer, offered an apt but simple metaphor. She likened spirituality to water, and religion to the cup that holds the water and carries it forward (2015). Religion is an important, if under-recognized, social determinant of health, a source of social support and social capital and a powerful regulator of individual and group behavior (Idler 2014).

Without question, religion’s influence on global health has been mixed. On the one hand, religious communities provide a sizeable proportion of health care in many regions (Brown 2014; Mwenda 2011), particularly for populations that are marginalized, neglected, and underserved by government institutions. The primary health care movement of the 1980s owes much to the influence of the Christian Medical Commission of the World Council of Churches (Bersagel Braley 2014). This movement emerged from a profound process of theological discernment and moral reflection, guided by a compassionate desire to alleviate suffering and to fulfill the mission of the ‘healing church’ in a post-colonial world (McGilvray 1981). Religious influences are also apparent in the ongoing dialogue between Dr. Paul Farmer – one of the most prominent global health leaders – and theologian Fr. Gustavo Gutierrez (Griffin and Weiss Block 2013). Their rich exchange highlights the ways in which Roman Catholic liberation theology, in particular, can inspire global health’s pursuit of social justice and ground it in a rich, nuanced, spiritual framework.

At times, religion has also impeded the global health agenda. In the words of Mother Teresa, the problem with the world – and I would add, with religion – “is that we draw the circle of our family too small” (Reifenberg 2013). By reinforcing in-group vs. out-group prejudices, religious beliefs have interfered with treatment and prevention of HIV/AIDS and stigmatized those who suffer from it (George Dalmida and Thurman 2014). Religious ideology also has been used as the justification for refusal to immunize children against transmissible, deadly infectious diseases and to oppose family planning and reproductive health services. For all of their benefits, short-term mission trips, both by health professionals and volunteers, have been criticized for lack of long-term effectiveness, cultural insensitivity and naivety and for undermining rather than strengthening local health systems (Brown 2014).

In response to this mixed history, modern global health, which seeks health equity for all peoples, remains wary of religion and its potential for divisiveness. The field is largely secular, pluralistic, science-based, results-oriented, structurally complex, and predominantly funded by the public sector. In this context, open expression of one’s religious beliefs or spiritual values is not encouraged. Indeed, spiritual concerns are often considered a distraction to the vastly more important and practical work of health equity and social justice.

A few years ago, outside the work setting, I began asking my colleagues from the US Centers for Disease Control and Prevention (CDC) to describe, in one word, why they worked in global health. Typically, they became pensive, their voices dropped low, and they responded with words such as “compassion,” “concern,” or “caring” – even “love.” I was struck by their vulnerable responses to this simple question and by the consistent themes that emerged – about which we had never spoken. Many described their decision to enter global health as an expression, an outward manifestation, of deeply-held values that give their life meaning and purpose. Some referred to it as a calling.

To better understand the role of spirituality, broadly defined, in the practice of global health and to identify factors that influence and limit its expression in global health settings, I initiated conversations and conducted informal, open-ended interviews on these themes with more than three hundred global health leaders, students, practitioners, physicians, and nurses from 2010 to 2014. The conversations took place within the context of meetings on neglected tropical diseases at the Geneva headquarters and regional offices of WHO, The World Bank, and CDC; at annual meetings of the American Society of Tropical Medicine and Hygiene; in conferences, classes, and seminars at Emory University (Atlanta), George Washington University (Washington, D.C.), Washington University (St. Louis), the University of Notre Dame, and Children’s Hospital of Philadelphia; and during a September 15-17, 2010 meeting on compassion in global health at The Carter Center, in Atlanta (Task Force for Global Health 2011).

## Spiritual themes and challenges

Four interrelated spiritual themes emerged through these conversations, four areas of challenge that global health practitioners identified as contributing to a lack of connection with the values that drew them to the field. I describe these challenges as: 1) compassion at a distance; 2) dichotomous thinking; 3) conspiracy of silence; and 4) compulsion to save the world.

### Compassion at a distance

The very nature of global health presents specific challenges to the cultivation and expression of compassion. What does it mean to have compassion for entire populations from whom one is separated by great geographic, cultural, or economic distances? Many of the practitioners I interviewed spoke of compassion as a motivating force for their having entered global health, but they also expressed a sense of disconnection from this compassionate impulse in their day-to-day work.

Conceptual models of compassion described by Batson (1987), Eisenberg (2002) and Halifax (2011) provide useful frameworks for understanding this disconnect. At the risk of oversimplification, these models suggest that compassion involves three elements: awareness of suffering (cognitive attunement); empathy (emotional attunement); and action (to relieve suffering). First, a compassionate response requires the stability and clarity of mind to recognize suffering as suffering. In global health, the perception of suffering is often based on data or indices rather than on face-to-face encounters with affected individuals. Especially for conditions that, at the individual level, are subclinical, hidden, or chronic such as micronutrient deficiency (Sommer 1996) or infection with soil-transmitted helminths (Hall 2008), the link with suffering can seem abstract.

Former Director of the CDC, Bill Foege, highlighted this fundamental challenge of ‘compassion at a distance’ in a speech delivered at CDC in 1984: “If we are to maintain the reputation this institution now enjoys, it will be because in everything we do, behind everything we say, as the basis for every program decision we make—we will be willing to see faces” (1984). This was an extraordinary message for a public health institution, responsible for the health of populations, not individuals. CDC’s reputation would depend not on programmatic effectiveness, measurable outcomes, or epidemiologic prowess but on compassion – the willingness of its employees, collectively, to see the faces of suffering.

Second, compassion requires empathy, the capacity to feel the pain of the other. Disembodied data are seldom sufficient to stimulate emotional arousal. For those in global health who work at the population or policy levels, this emotional distance can be difficult to overcome. The opposite is true for those who serve in situations of overwhelming, extreme suffering coupled with inadequate resources and, at times, personal danger. For workers in refugee, disaster, or relief settings or in zones of conflict, instability or violence, the emotional arousal can be too intense, triggering feelings of helplessness, fear, inadequacy, and anger. In these situations, the threats to compassion are emotional overload and personal distress, which lead to responses of fight, flight, or freeze rather than to compassion.

Third, compassion demands action. The tools of global public health, the means through which compassion is expressed and suffering relieved, often are not direct and personal as in the clinical setting but organizational in nature, e.g., program planning, budgets, grants, protocols, training, evaluation, and logistics. Large global health programs are implemented through complex partnerships. Negotiating organizational policies, politics, and agendas, while necessary to mobilize resources needed to address the suffering of populations, can distract and divert the attention of global health workers away from the faces of suffering. The empathic signal easily fades to the point of extinction. This can occur within a relatively short period of time; many of the young professionals with whom I spoke, just a few years out of public health school, expressed disillusion, a loss of idealism, and estrangement from the people they intended to serve.

### Dichotomous thinking

A second spiritual challenge in global health is dichotomous thinking, the tendency to view the world in mutually exclusive ‘either-or’ categories such as sick or healthy, dead or alive, case or control. This kind of binary classification has proven extraordinarily powerful in epidemiology, the scientific discipline that undergirds and informs global health. Yet it also has its limitations. Dichotomous thinking inhibits our capacity to appreciate paradox and to solve complex problems (Palmer 2008). It also is fundamentally inconsistent with the lived experience of many in global health for whom the rigid categories of here-there, local-global, and us-them no longer serve.

Throughout history, contemplatives from the spiritual and religious traditions have pointed to the value of non-dual or non-judgmental awareness, which transcends rigid, mutually-exclusive, dichotomous categories (Palmer 2008; Zajonc 2009; Rohr 2009; Matthiessen 1985). For those of us trained to think epidemiologically, the notion of non-duality seems nonsensical, or at least non-scientific, as epidemiology typically adheres to a Newtonian world view. In contrast, the sciences of physics and consciousness studies have had to move beyond subject-object dualism to make sense of their experimental data (Zajonc 2009; Dalai Lama 2005; Wallace and Hodel 2007).

The capacity for non-dual awareness, for holding ‘the faces and the numbers’ *at the same time,* may be precisely what is required for global health leadership (Foege and Rosenberg 1999). In a speech in 2012, Bill Foege alluded to the geographic non-duality of global health when he said, “Everything is local and everything is global. Global health is not ‘over there’ – it’s right here” (Bill Foege, personal communication, Task Force for Global Health in Decatur, Georgia, April 26, 2012). Hunter and Fineberg (2014, 1755) recently described global public health as “a global tapestry of influences,” within which “the individual patient encounter is a local event, and global health institutions may constitute a patchwork of entities.” Thus, for global health to realize its promise of transforming the suffering of entire populations, its practitioners must have the capacity to remain in relationship with individual human ‘faces’ while immersed in the numbers, to see the whole and the parts simultaneously and to move seamlessly from local to global and back again. These non-dual skills are taught and nurtured in the contemplative traditions of many religious and spiritual pathways but not in schools of medicine, nursing, or public health.

### Conspiracy of silence

One of our most persistent dichotomies is the divide between the inner self – and that which gives life meaning and purpose – and our action in the world. We who work in global health tend to quarantine our spirituality, never collectively acknowledging its animating power. This creates an inner dividedness, a silent form of interior suffering. The reasons for such a conspiracy of silence are not entirely clear. As noted earlier, given the immensity of suffering and disease in the world, any focus on one’s own inner life can be considered a luxury, a distraction to the more important work of global health. Grounded in the science of epidemiology, global health is results-oriented and distinctively secular. In this context, public expressions of personal values are not encouraged. Yet, in the words of theologian Gustavo Gutierrez (2003), we must “drink from our own wells” of spirituality if we are to sustain effective action.

A recurrent theme in my conversations was the importance of a personal encounter with a particular individual – for example during an international service learning project or a medical student clerkship abroad – in stimulating a decision to enter global health. The stories of these encounters hold tremendous power to motivate, shape, and sustain an entire career, yet they are rarely shared collectively. When space is opened for such conversation, the response can be powerful. On December 7, 2011, a symposium on compassion and global health was held at the annual meeting of the American Society of Tropical Medicine and Hygiene (Addiss and Rosenberg 2011). During the discussion period, members of the audience – distinguished experts in tropical medicine – lined up to share their personal stories. A comment I later received from a program officer who attended was typical: “The symposium was a milestone for me. I never thought the American Society of Tropical Medicine and Hygiene would approve a symposium on such a topic. It's also been an individual journey as I try to reconnect with my own sense of compassion and motivation for this work. I hope that we find ways to advance the conversation.” Like this program officer, many students and young professionals with whom I spoke expressed a strong desire to break the taboo of silence around the spiritual values that inspire them and undergird the field of global health.

### Compulsion to save the world

A fourth spiritual challenge that surfaced in my conversations is more subtle and, therefore, more difficult to recognize than the others. It represents what Carl Jung would describe as the shadow side to the sense of calling, mission, and purpose that many global health practitioners find in their work.

A career in global health offers the opportunity to ‘do good’ on a large scale, to have a measurable impact on the suffering of millions of people. The skillful and systematic application of relatively simple technologies can dramatically improve child survival, eradicate scourges such as Guinea worm disease and smallpox, and eliminate the debilitating effects of neglected tropical diseases that affect more than one billion of the world’s most economically impoverished people. Thus, a career in global health appeals not only to a compassionate impulse rooted in mature self-awareness but also to the ego’s desire for significance and identity. The sweeping vision and scope of global health presents a compelling case for those who might be seeking to ‘work out their salvation’ or to be affirmed as a good or caring person.

Linking one’s identity and self-worth to a field as vast as global health can result in a great deal of frenetic activity in the name of service to others. If not examined, this can be highly problematic, even though it is usually justified on the grounds of the tremendous need. While not referring to global health, Thomas Merton, a Cistercian monk, described this problem when he wrote: “To allow oneself to be carried away by a multitude of conflicting concerns, to surrender to too many demands, to commit to too many projects, to want to help everyone in everything is itself to succumb to the violence of our times” (1965).

How does excessive self-identification with one’s work affect global health practitioners? First, it can adversely affect relationships with family and loved ones. An AIDS activist, Geoffrey Knox, wrote to me the day after an evening conversation saying, “I don’t think I had verbalized to anyone before the disconnect I sometimes feel between the compassionate nature of my work, all with nonprofits doing extraordinary public health and social justice work, and my avoidance of dealing directly and compassionately with friends and family who are in psychological or physical distress.”

Second, the choices and drive required to climb the ladder of influence in global health can inhibit the ability to perceive the shadow side of one’s commitment. With such a significant personal stake in one’s work, it can be difficult to welcome criticism or to acknowledge when the outcomes of one’s projects are not as one hoped. Clinging to a particular outcome is inconsistent with a mature understanding of compassion. It can lead, instead, to so-called ‘selfish’ pro-social behavior, which addresses the needs of the ‘helper’ more than the intended recipients of one’s care.

Third, one of the major challenges in global health is the imbalance of power and resources between ‘donor’ agencies, whether governmental or private, and ‘recipient’ communities. Those on the donor side exacerbate this power differential when they are convinced of the righteousness of their cause, program, or intervention or over-identify with its success. They unwittingly contribute top-down ‘solutions’ to problems that may not be appropriate for local communities. Too often, priorities in tropical medicine and international health have been driven by donors, foreign policy, or other factors (Farley 1991; Gow 2002).

Finally, excessive self-identification with the righteousness of one’s cause contributes to an inability to come to terms with unintended harmful consequences of one’s actions. Apology for medical errors and misadventures has become more commonplace in medicine in recent years (Wood and Star 2007). Unfortunately, apology is still a rarity in global health. Global health does not have a tradition of honoring or publicly acknowledging those whose lives are lost or diminished as a consequence of well-intended interventions, as happened when mass treatment with ivermectin, intended to prevent blindness due to onchocerciasis, was associated with the deaths of those who happened to be heavily infected with the African eye worm parasite, *Loa loa* (Twum-Danso 2003). This makes it difficult to grieve loss and apologize for harm, both of which are necessary for inner healing and restoring of relationships.

## Discussion

Informal interviews and conversations with global health leaders, students, and practitioners suggest that while global health is rooted in spiritual values (Brown 2014), spiritual questions and concerns are rarely raised in global health discourse. In my interviews and conversations, four specific spiritual concerns or challenges consistently emerged. They are not unique to global health and certainly are not the only spiritual challenges for those in this field. Undoubtedly there are others, which warrant further exploration and attention.

The accomplishments of global health and its predecessors – tropical medicine, public health, and international health – are impressive. Infant mortality has declined and life expectancy has increased dramatically in the last century (Levine 2004). Diseases of neglected populations are attracting unprecedented attention and resources (WHO 2014). In light of these achievements – and in view of the pressing needs and immense suffering – is Merton’s caution about “surrendering to too many demands and committing to too many projects” irrelevant? Indeed, His Holiness the Dalai Lama, a powerful teacher on compassion, colorfully underscores the importance of action: “[Compassion] is not just a wish to see sentient beings free from suffering, but an immediate need to intervene and actively engage, to try to help “(2002, 225)…” A person who has attained stability in his or her compassion training … should now be out, running around like a mad dog, actively engaged in acts of compassion” (2002, 91).

The sheer scope of global health action in the world today is ‘mad-dog’ impressive. But the action to which the Dalai Lama refers arises from a sense of inner stability and spiritual groundedness – and this is often missing in global health. With our singular focus on systemic change and our drive to achieve measurable results, it is easy to neglect the relationships that sustain us and make the work possible. The fields of liberation theology (Griffin and Weiss Block 2013) and end-of-life care (Halifax 2008) offer important lessons to global health on the primary importance of presence, solidarity, and accompaniment in informing and guiding compassionate action.

These lessons are relevant not only to global health, but to many other fields and disciplines with global aspirations. Writing for a broader audience, Joanna Macy (2000, 135) said, “To heal our society, our psyches must heal as well. The military, social, and environmental dangers that threaten us do not come from sources outside the human heart; they are reflections of it, mirroring the fears, greeds, and hostilities that separate us from ourselves and each other. For our sanity and our survival, therefore, it appears necessary to engage in spiritual as well as social change, to merge the inner with the outer paths.” Indeed, the spiritual traditions consider integration and alignment of one’s spirituality and one’s work in the world as central to authentic living (Griffin and Weiss Block 2013; Palmer 1990).

Little is known about spiritual practices that sustain and inform those in global health. Some interviewees reported drawing on their religious faith to orient, ground, and give meaning to their work. Others spoke of the importance of meditation, time for reflection, and daily personal rituals. For global health practitioners whose tasks are largely administrative and who work in large organizations, trips ‘to the field’ to literally ‘see the faces’ are crucial for restoring a sense of connection and purpose. There, the impact and human dimensions of their work become more tangible, they become reacquainted with the faces of suffering, and the emotional resonance required for compassion is rekindled. Photographs from these trips are placed in their offices to remind them for whom they work.

This inquiry focused on spiritual challenges of individual global health practitioners, but the themes that emerged are also relevant for global health institutions. In particular, compulsion to save the world can be observed in the way global health policies are formulated and programs implemented. Messianic fervor is not limited to religiously-motivated individuals and institutions; government agencies are also susceptible, particularly when national interest or the reputation of their leaders is at stake. During the early days of tropical medicine, a precursor of modern global health, religious and nationalistic motivations comingled as medical officers from Europe and North America took up the ‘white man’s burden’ to address the challenge of tropical diseases (Farley 1991). The ongoing influence of compulsion to save the world in modern global health is suggested by programs that, while unquestioningly delivering health benefits, do so in a way that engenders charges of neo-colonialism (Sastry and Dutta 2012). Further, when national security is on the line and governments feel threatened or fearful, compulsion to save the world can easily devolve into compulsion to save *my* world. A recent publication from the Center for Strategic and International Studies extolled the advantages of global health engagement as “a key tool” for the US Department of Defense (Daniel and Hicks 2014) – which, in my view, undermines the core principle of health for all peoples.

How might a more open dialogue and a more explicit awareness of our individual and collective spiritual roots contribute to global health? First, it might offer a vocabulary and language to encounter, understand, and more firmly embrace global health’s fundamental values of interconnectedness and compassion. Second, familiarity with the contemplative traditions might help global health practitioners experience and understand the non-dual world in which they live, move, and have their being, and improve their capacity to see ‘faces and numbers’ at the same time. Third, spirituality might play a critical role in grounding global health in the daily practice of compassion. Practices that foster non-referential compassion, i.e., unbiased compassion without a specific object (Halifax 2011) may be particularly useful to help practitioners remain grounded in compassion when working with populations. Fourth, such a dialogue could foster the self-awareness necessary for global health practitioners to recognize, and surface, unhealthy or shadow dimensions of their work. Finally, a deeper grounding and acknowledgement of spiritualty can help provide a more robust ethical and moral framework (Reilly 2008; O’Connell 2009) from which to negotiate the complexities of modern global health and to recognize and encounter the forces that act contrary to its core values, such as nationalism, militarism, and greed. Such an approach to spirituality could also serve to foster much-needed trust and understanding between secular governments and faith-based groups engaged in global health.

Professor john a. powell has eloquently described the bi-directional relationship between spirituality and social justice and noted that the world is both “deeply spiritual and deeply secular” (2003, 127). The pursuit of social justice, which lies at the heart of global health, has much to contribute to the development and practice of spirituality. Indeed, global health’s commitment to relieve suffering on a massive scale and its insistence on rigorous measurement to verify the effects of its interventions are attracting to its ranks a small but increasing number of students trained in theology and religion.

This exploratory inquiry has many limitations. Global health is a massive, diverse field; the ideas, concerns, and thoughts of the people with whom I spoke – a relatively small convenience sample – may not be representative of the field as a whole. The interviews and conversations were unstructured, conducted ‘in the corners’ of meetings, conferences, and speaking engagements over a five-year period. However, as an exploratory inquiry, they surfaced a strong interest in – and hunger for – bringing themes of compassion, spirituality, and core values into global health discourse (Center for Compassion and Global Health 2015). Doing so could ground and strengthen the field itself, help animate and support those who dedicate their lives to it, and improve their capacity to accompany, and stand in solidarity with, those they seek to serve.
